# Health@Home Moves All About the House!

**Published:** 2016

**Authors:** Gail R. CASPER, Patricia F BRENNAN, Catherine ARNOTT SMITH, Nicole E. WERNER, Yuqi HE

**Affiliations:** a School of Nursing; b Wisconsin Institute for Discovery; c School of Library and Information Studies, College of Engineering; d University of Wisconsin-Madison

**Keywords:** personal health information management, self-care, information technologies, home context

## Abstract

It is now well recognized that patients play an important and active role in self-care and disease management, and many of these activities happen in their homes. Information technologies to support such care might be better used if they were designed taking into account the physical context of the home and the health information management needs of the residents. We conducted home-based interviews of 20 adults including an extensive analysis of their personal health information management (PHIM) tasks. Here we present these task descriptions, locations of their performance, and distribution across space and time. Implications for the informatics community include accommodating the distributed nature of tasks in the design of consumer technologies.

## 1. Introduction

The ‘home as a site for care’ has emerged as an important target for consumer health informatics tools, and there is a rush of smart appliances^1^, interactive health technologies^2^, home care apps and connected self-monitoring devices. Yet innovations intended for “home care” or “self-monitoring” are often designed with little attention to the physical characteristics of the place where such devices are used, resulting in little guidance on strategies to address and accommodate physical characteristics of the home including its spatial orientation, furnishings, and personal objects. The purpose of this paper is to explore personal health information management (PHIM) in the home, with attention to the extent to which the tasks are distributed over time and space. We believe such findings provide new insights to the design and the deployment of consumer health informatics innovations for self-care.

The need to explicate the impact of the physical context of the home on PHIM arises from three trends: 1) evidence that physical contexts shape health outcomes^3^; 2) the rise of home monitoring, health apps, and other technologies to support PHIM, and 3) the relative lack of guidance on the design of consumer health informatics innovations^4^. This paper, and the larger project from which it emanates (HS 22548, *vizHOME*), addresses trends 2 and 3: to accelerate the design and deployment of context-appropriate consumer health informatics tools to support PHIM.

PHIM encompasses a broad range of behaviors from seeking diagnostic information on the web to self-tracking. To define the field of exploration for this study, we adopted the three areas in which PHIM is being investigated as identified by MacGregor and Walthen^5^ 1) *storage and organization of health information in electronic or paper form; 2) tracking or recording observations such as symptoms or calorie intake; 3) seeking health-related knowledge or information.*

In Project HealthDesign^6^ we observed that not all PHIM tasks involved use of a physical artifact; thus we include as PHIM tasks the cognitive and decision making tasks used for self-monitoring (e.g. selecting an insulin dose based on a glucometer reading). Thus PHIM comprises a suite of behaviors and cognitive strategies used by an individual to record, organize, act on, store, retrieve, or coordinate information related to health and health care.

The growing literature on PHIM tasks in the home has focused on either self-report by patients or laboratory observations of prompted task performance^2,7^. These approaches attend to the psychomotor aspects of PHIM but they provide minimal description of the physical space of the home where tasks occur. However, we know that aspects of the home such as privacy, lighting, or storage access influence PHIM and the usefulness of devices to support it^8^.

In earlier work exploring PHIM in the home, participants were asked to “show me how you do …” without specific attention to context^2^. Our group employed a modified contextual inquiry to explore the spatial dimensions and natural trajectory of PHIM tasks from the perspective of the person in his or her home. Participants identified tasks that were most important to them; then described or demonstrated the steps of each task and where they performed them in a typical day. In addition, we believe this approach better illuminated cognitive aspects of PHIM, such as triggers and recall, as well as how visual cues in the home prompt PHIM tasks.

## 2. Methods

### Parent Project

The data we report represent the first phase of the parent vizHOME project: exploring PHIM task performance in homes. We had approval from the Institutional Review Board for this phase of the study. Participants were compensated for their time.

### Setting and sample

The target population was community-dwelling adults who reported having diabetes and lived in homes in urban, suburban or rural regions of a mid-western state. Four types of homes were targeted (detached, semi-detached, multi-unit and mobile) to represent a range of households. Adults with diabetes were selected as informants because they must regularly engage in a number of self-care activities involving PHIM tasks, and the disease affects many aspects of daily life, such as food selection and exercise needs.

### Data Collection and Data Analysis Procedures

Similar to Dabbs and colleagues^2^, we employed a modified contextual inquiry process involving three home visits to each participant, each visit lasting 2–3 hours. Contextual inquiry is an unstructured but purposeful interactive process situated in the person’s natural environment. We employed a work systems approach to characterize the PHIM tasks and a consolidation strategy for data analysis.

Interviews were audio-recorded and were conducted by two study team members, an interviewer and a notetaker. Participants responded to questions about their health concerns, self-monitoring and self-management practices. A schematic map of the home was created to highlight layout and focal areas for PHIM. Participants identified specific PHIM tasks in the locations where they performed them when possible. All instruments and forms are available from the authors.

Additional data collected included general location of the home, global health rating, perceptual or mobility limitations, others living in the home, presence of pets and a home clutter rating. We also obtained full-scale 3D scans of the interior of each home. Data consolidation processes applied the Health@Home model^9^ to characterize information management. Finally, we reviewed home maps to facilitate reconstruction of task activities across space and time.

## 3. Results

We interviewed 20 people in their households during 2014–2015. The mean age of participants was 59 (sd=12), 65% were white, 70% were females, and 25% lived alone. All participants had a cell phone, a land line or both; all but one household had a laptop or a personal computer. We identified over 100 different tasks, and explored 60 in depth (3 per participant). Table 1 provides examples of PHIM tasks reported by the participants.

We now provide three detailed examples of PHIM tasks, locations and time sequences reported by participants to illustrate this point. The schematic maps of the homes highlight distribution of tasks in the home as well as over time by solid vs broken arrows. See Table 2.

Findings demonstrated that people perform PHIM tasks in multiple areas of the home. This suggests a distributed nature of even apparently simple tasks, such as taking a medication. These vignettes illustrate the distribution of the location and of the timing of PHIM task performance within individuals and across households. By consolidating data from the interviews and the maps, we discovered that PHIM task steps occur throughout the day and the home; most commonly in the kitchen, living room and bedroom.

We examined the extent to which PHIM task performance was distributed in space and time across all participants. We analyzed a total of 60 tasks, 20 medication management tasks, self-monitoring tasks and information management tasks. With only one exception, tasks related to medication management occurred not at a single point in time, but as a discrete set of steps unfolding over long intervals (95%) and in several different spaces in the home (90%). Similar variability in space and time was observed with information management tasks (75–80%) and self- monitoring tasks (80–55%).

## 4. Discussion

There is no one place or time of day that PHIM tasks occur in the home. We explored a total of 60 different PHIM tasks identified in the course of three home visits with 20 people recruited for a larger study of how the home context influences PHIM. We expected that PHIM tasks, such as medication management, would happen in a specific place at a given, well-circumscribed time. This is evident in the case of Participant A, who has a specific time of day and specific space where she does her blood glucose monitoring. Even in this single act, however, there are interruptions and additional activities in additional spaces. Conversely, Participant L, who takes many medications during the day, starts planning and sorting actual pills early in the morning. He does not use a chart or record, but relies on memory. He distributes small containers of these pills throughout the house to intersect in the place he anticipates being at that time. We characterize PHIM tasks that unfold over space and time as “distributed”.

Participant L used strategically placed objects, like a pill bottle, to serve as a visual cue to action. Palen and Aaløke’s^7^ description of the structures of ‘pill boxes and piano benches’ was echoed in the self-made solutions we uncovered in this study. From TV shows serving as cues to take a medication, or the color-coding on a kitchen calendar to signify types and timing of appointments, to having a refrigerator in the bedroom to enable taking medication after retiring - the individualization of routines customized to the home, even specific areas within the home, was apparent. This finding underscores the importance of design informed by understanding how and where patients actually perform tasks in their homes – not how they tell us they would perform them or even how they show us when in a hospital or clinic.

Even when people had access to technology, there was less reliance on computer and communications technologies, such as cell phones, laptops and/or desk top computers for PHIM. Participant J employed an extensive, redundant set of paper diaries to keep track of clinic visits, a very common type of PHIM. Here the artifact is not displayed and does not provide any visual cues to action, but rather serves as an archive. It is notable that the various diaries are stored in different places in the home, some being more accessible, others affording more privacy. Our findings suggest that PHIM is supported by three interrelated resources: 1) cognitive functions (memory and recall, 2) health-related objects (pill bottles and organizers) and 3) artifacts (diaries).

### Limitations

The work presented here relies on descriptions of behaviors, actions and visual cues to infer motivation and intention. In addition, although participants reported that a part or all of some tasks occurred outside of the home, we did not capture how they occurred or were distributed over spaces outside of the house.

### Conclusions

Our work complements emerging work in distributed cognition suggesting a rich environment for novel health information technologies. We have transformed consumer health informatics through a patient work framework: connecting patients to context.

## Figures and Tables

**Table 1:** Personal Health Information Management Task Examples

Medication Management	Self-Monitoring	Information Management
Manage & execute med schedule Restock7-day pill organizer Prepare & take daily meds	Monitor & track blood sugar levels Monitor blood pressure & cardiac status Assess, treat & monitor headaches	Plan clinical appointments Search for, use, & store info Journal about health conditions or emotional state

**Table 2:** Vignettes and Home Layouts

Each day at 9:30am, Participant A retrieves her glucometer and log book from the dresser in the spare bedroom. She brings it to the kitchen table where she checks and records the reading. If she wants help interpreting the reading or advice on what to do, she goes to telephone in the living room to call a friend who has diabetes. When done, she returns the glucometer to her bedroom. She repeats this procedure before her evening meal. She relies on memory and routine to perform these tasks.	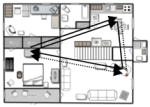
Participant J has four to five appointments with different health care providers each week at varying times of the day. She uses both a traveling planner and a main planner to record all her appointments. As she leaves an appointment she records upcoming appointments in the travel planner, which she stores in her backpack. When she returns home, she transfers the appointment information into her main planner which she keeps under her bedside table. She refers to this main planner on a daily basis.	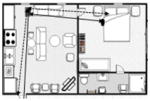
Every morning before breakfast, Participant L counts out his morning and evening pills from their individual bottles that he stores on his bedroom dresser. He leaves his evening pills on the dresser and takes his morning pills with him to the kitchen where he obtains an aspirin from a cabinet; he then ingests all pills at the dining table. He has another pill that he takes with dinner; he keeps this medicine bottle on the dining table as a reminder to take it. Around 9pm, when his wife goes to bed, he retrieves his evening pills from the bedroom dresser and brings them to the kitchen counter. He takes them around 11pm. He primarily uses environmental cues and diurnal events to perform these tasks.	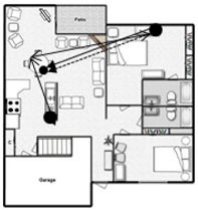

## References

[1] Mizukura I, Tamura T, Kimura Y, Yu W (2009). New application of IEEE 11073 to Home Health Care. Open Med Inform J.

[2] Dabbs ADV, Myers BA, McCurry KR, Dunbar-Jacob J, Begey A, Dew MA (2009). User centered design and interactive health technologies for patients. CIN.

[3] Payne RA, Abel GA, Buthrie B, Mercer SW (2013). The effect of physical multimorbidity, mental health conditions and socioeconomic deprivation on unplanned admissions to hospital: a retrospective cohort study. CMAJ.

[4] US AHRQ (2012). Designing Consumer Health IT: A Guide for Developers and Systems Designers.

[5] MacGregor J, Walthan CN (2014). ‘My health is not a job’: a qualitative exploration of personal health management and imperatives of the ‘new public health’. BMC Pub Health.

[6] Brennan PF, Casper GR (2015). Observing health in everyday living: ODLs and the care-between-the-care. Pers Ubiq Comp.

[7] Palen L, Aaløkke S (2006). Of pill boxes and piano benches: “Home-made” method for managing medication. CSCW.

[8] Demirkan H, Olguntürk N (2014). A priority-based “design for all” approach to guide home designers for independent living. Archit Sci Rev.

[9] Moen A, Brennan PF (2005). Health@Home: The work of health information management in the household: Implications for consumer health informatics innovations. JAMIA.

